# The Nervous Accidents of Syphilis

**Published:** 1875-01

**Authors:** John Ben. Stonehouse

**Affiliations:** Formerly Assistant Medical Superintendent Sanford Hall, Flushing, N. Y., one of the Attending Physicians at the Albany Hospital Dispensary Member of the American Association for the Cure of Inebriates, etc., etc.; 373 Clinton Avenue, Albany, N. Y.


BUFFALO

### Medical and Surgical Journal.

VOL XIV. JANUARY, 1875. No. 6.

### Original Communications.

:o:

ART. I.—*The Nervous Accidents of Syphilis. A Lecture delivered to the Surgical Class of the Albany Medical College, November* 21, 1874. By JOHN BEN. STONEHOUSE, M. D., formerly Assistant Medical Superintendent Sanford Hall, Flushing, N. Y., one of the Attending Physicians at the Albany Hospital Dispensary, Member of the American Association for the Cure of Inebriates, etc., etc.

GENTLEMEN—I shall be compelled to limit myself this evening simply to the statement of facts and those of the most practical character, although my subject gives ample opportunity for theorizing. "The multiple character which is so frequently to be observed in the more serious nervous affections consequent upon syphilis renders well nigh impracticable any attempt to group them in distinct classes."* For this reason the order in which I treat of these affection will be entirely arbitrary.

PARALYSIS.—Constitutional syphilis may produce paralysis of almost any kind. Dr. Otto Braus reports 130 cases of brain syphilis. In 34 cases paralysis of optic nerves occurred. In 27 facial paralysis. In 22 hypoglossal. In 17 paralysis of bladder. In 15 of intestines. In 31 cases hemiplegia occurred, and in 8 paraplegia. Paralysis of a single limb was observed in 18 cases.

* Buzzard Clinical Aspects, etc., 1874, London.

Paralysis is usually incomplete and irregular in distribution and course. The attacks may be unusually transient but they generally persist for some time. We often find instead of actual paralysis a very high degree of weakness or paralysis of energy, sometimes accompanied by mental disturbances, at others uncomplicated; often this condition becomes one of actual paralysis. Contraction may occur in connection with syphilitic paralysis, but it is quite rare. Muscular atrophy very often accompanies syphilitic paralysis. Dr. Anstie has reported a case of syphilitic paralysis with unusually rapid muscular atrophy. Middle age is the period of syphilitic paralysis; cases characterized by repeated relapses after prolonged periods of immunity occurring during this period, and non-hysterical are seldom due to any cause save syphilis. Young adults free from cardiac and renal disease should be suspected sooner than patients of greater age. "I have little hesitation in stating my conviction that putting aside cases of injury, hemiplegia or paraplegia occurring in a person between 20 and 45 years of age, which is not associated with Bright's disease, nor due to embolism (from disease of cardiac valves) is in at least 19 cases out of 20 the result of syphilis."

Several authors have of late, in the European medical periodicals, reported cases of pseudo-paralysis, an inability to move certain limbs, most frequently the leg, arising in part from separation of the epiphyses, and in part from pain. It is the result of hereditary syphilis. The changes begin in utero, and (in addition to the osseous and muscular lesions) are accompanied by abscesses at joints. The pseudo paralysis is in no proportion to the visceral and cutaneous lesions.

HEMIPLEGIA.—Hemiplegia is remarkably more frequent than paraplegia. Dr. Hughlings Jackson relates the following causes of hemiplegia in syphilis:

1st. By syphiloma of the surface of brain causing epileptic attack and hemiplegia; this is the epileptic hemiplegia of Dr. Todd, a very common mode of origin of hemiplegia from gumma is this. The syphiloma causes unilateral convulsions, (of which we shall have more to say hereafter). These are followed by paralysis usually of the side opposite to the convulsions.

2d. By the growth of a nodule in the motor tract—the hemiplegia being very slow in its development.

3d. By disease of the middle cerebral arteries or their branches.

4th. By the growths in the channel of the artery, the formation of a plug and consequent hemiplegia usually followed by cerebral softening. There is no preference of syphilitic hemiplegia to either side of the body. The symptoms are peculiar; there is marked irregularity in the loss of motor power, and a wonderfully random succession and association of phenomena. The paralysis is usually slow in its onset. Frequently accompanying the paralytic symptom we have anæsthesia or hyperæsthesia of the opposite side, and sometimes of only one limb. Aphasia occurs quite frequently, not necessarily in company with right hemiplegia, for as will be noted hereafter syphilitic diseases of the cerebro-spinal axis are seldom the effect of a single lesion. Hemiplegia occurring in the course of primary mental disease is extremely unfavorable. In paralysis we may expect recovery if we can exclude thrombosis and softening, and if too long a time has not elapsed. The paralytic symptoms frequently become chronic and general, and accompanied by mental disturbances more or less marked. After hemiplegia has subsided chorea not unfrequently attacks the patient. Various other motor disturbances follow the disappearance of paralytic symptoms, characterized by rapid onset, succession and cessation. In the diagnosis of syphilis as a cause of hemiplegia we must exclude other causes such as embolism from cardiac disease; the age and general health of the patient, and the mode of onset must also be considered.

Paralysis of Cranial Nerves.—Paralysis of individual cerebral nerves are comparatively frequent. In Braus' 82 cases there occurred 34 cases of paralysis of optic nerve, 27 of facial, 22 of the hypoglossal. We frequently find blepharoptosis produced by paralysis of the levator palpebræ muscle supplied by the 3d pair and strabismus — external from paralysis of the internal recti supplied by the 3d pair or mortores oculorum — internal from paralysis of the external recti supplied by the 6th pair, the abducentes, very frequently occurs in syphilitic cerebral disease. Excepting the nerves of special sense, the 3d pair is most commonly affected, although its affection is more common in non-syphilitic disease; the 6th nerve is affected with next greatest frequency. Paralysis of the 4th—the pathetici—shown by an inability to rotate the eyeball, and consequent double vision is seldom observed in syphilitic disease. Syphilis never produces paralysis of the 7th pair—facial or portia dura. Hence we see how important in the diagnosis of cerebral disease, the study of the affections of the cerebral nerves may be made. Their paralyses are seldom caused by non-syphilitic disease, and almost invariably occur among the tertiary phenomena. The 3d nerve is however an exception to this statement. When these palsies do occur in the secondary period they are commonly circumscribed of a single nerve or its branches—occasionally a palsy of both the 3d and 6th pairs is observed. Fournier has never seen a palsy of the 4th pair during the secondary period. The palsies of these nerves generally appear successively, and are liable to remarkably quick and frequent occurrence. The "facial hemiplegia" just mentioned as consisting of palsies of both the 3d and 6th nerves is the earliest to appear, usually occurring during the 4th, 5th or 6th month after infection. Palsies of cerebral nerves commonly complicate syphilis from attacks which are unilateral and accompanied by mental derangement. Paralysis of the optic is often a precursor or concomitant of syphilitic mental disease. And here I must warn you never to form an opinion of a case of cerebral disease until you have submitted the patient to an ophthalmoscopic examination. Optic neuritis significant of gross lesion of the intra-cranial space does not *necessarily* affect vision in the least, and its presence is so valuable as an aid to diagnosis that we should never presume its absence on account of perfect sight.

PARAPLEGIA.—Syphilis produces paraplegia with less frequency than hemiplegia from the same source. The tendency, according to Lanceraux, of most syphilitic spinal disease is to paraplegia. Bazin says that two-thirds of the cases of paraplegia are syphilitic. In Braus' 82 cases of cerebral paralysis we find paraplegia occurring eight times. Syphilitic paraplegia may occur from specific disease of the vertebral bones—from gummata, myelitis or from softening of spinal cord. In parapleg'a we find the same marked regularity in the loss of mortor power which we noticed in speaking of hemiplegia. Paraplegia is often incomplete, and when complete is more expressed on one side than the other. It generally extends to the bladder and rectum. Its symptoms are generally slow and halting. Syphilitic paraplegia usually occurs in early adult life or early middle age. At this period non-syphilitic degenerative, non-inflammatory softening is least likely to occur.

Traumatic paraplegia of course gives no trouble in diagnosis We must diagnose rheumatic paraplegia by the circumstances of its origin and the course of the affection.

Disturbances of Co-ordination.—We often find in the victims of constitutional syphilis disturbances of the co-ordinating power. There is often a tendency to perform gyrating movements as described by Neumann, (Wien. Med. Halle, IV., 2, 3, 1863, Schmidt's Jahrbucher t. 119, p. 166.) Examples of inability to walk in a straight line have been collected by Lagneaux. Lanceraux (Sydenham edition) Obs. L. reports a case where the patient inclined involuntarily towards the left. In his daily trip to the hospital he took the left sidewalk as it was the only one upon which he could remain.

Dr. Broadbent reports a case of locomotor ataxia which he attributes to meningitis, possibly syphilitic, along the posterior aspect of the cord, and also speaks of the posibility of multiple syphilitic deposits situated in sufficient numbers in the posterior white colums to impair spinal co-ordination. While in attendance at the Albany Hospital Dispensary I had under my care three women who exhibited all the symptoms of locomotor ataxia. They all presented an unusual want of fixity in the symptoms. In one case I have been able to trace a marked syphilitic history, and I am strongly suspicious of the other two cases. This would explain what is otherwise remarkable—so large number of cases among females in so short a period of time. It is not uncommon in marked syphilitic nervous disease to find a marked tremor. Several cases of this kind have been noted by Schutzenberger, and quoted by Lanceraux. This trembling may be simple and general or local, as the twitchings of muscles and startings of limbs which are very frequently precursors of syphilitic cerebral disease. These symptoms may precede or accompany the mental symptoms of cerebral syphilis, but are never observed after paralysis has set in. Occasionally after the disappearance of the paralytic symptoms, chorea has been noticed to occur; cases of hemichorea are reported by several French authorities, but it is seldom that the symptoms are so general, they usually are quite limited.

Epileptic and other Convulsions.—Convulsions dependent upon a specific causation may be produced in several ways; by gumma of the meninges or surface of the convolutions, by inflammatory deposit in similar localities, by disease of the cerebral arteries, as in non-syphilitic epilepsy, (Lanceraux says "Syphilitic disease of the arteries may interfere with the cerebral circulation only sufficient to impair nutrition to a degree permitting of discharges of nerve force as is frequent in epilepsy of advanced life.") By lesions of cranial bones, (Ricord speaking of the action of the syphilitic osseous affections on the neighboring parts says, "Another consequence of the species of compression is epilepsy. * * * The fits commonly seize the patient when the osseous growth producing the compression gets more considerable and irritating.") By albuminuria; this is not an unfrequent occurrence. A typical case may be found in Lidell's "Apoplexy." By reflex action; this may be the result of gumma or other growth in the periphery or of irritation arising from ulceration or erosion of portions of the posterior fauces, pharynx or larynx. I believe I have seen a number of case, not only of hysterical and epileptiform attacks produced in this way, but of almost every other variety of syphilitic nervous affection. Dr. Dewees of New York, in a paper on the paralytic and convulsive affections of cerebro-spinal system in the American Medical Monthly some years ago, first to my knowledge, called attention to this mode of causation. We commonly find a state of unilateral convulsions, in syphilitic cerebral disease. This was first noticed by Dr. Bright in 1835. Dr. J. Hughlings Jackson has of late called special attention to this affection. The most important symptom is unilateral convulsion unattended for the most part, with loss of consciousness. The convulsive movements may vary in degree from a slight twitch or stiffening to the most violent agitation, and may be accompanied or preceded by sensations of various kinds. The starting point is usually constant in a given case, and very frequently this will be the thumb and index finger. At one time it will be confined to the upper extremity, at another it may invade the entire lateral half of the body, traveling up the arm to the shoulder and face, and down the legs, becoming bilateral when the nerve nuclei of the two sides are associated. Sometimes the agitation and accompanying sensations are followed immediately by general convulsions and loss of consciousness. Some times the limbs convulsed are left paralyzed for a time from the exhaustion of the nerve force. A much more permanent paralysis however, often follows on the side opposite to the hemi-spasm, when the hemi spasm is on the right side, and especially when the face or tongue is the starting point, temporary loss of speech is very common. After these convulsions have for sometime existed, optic neuritis may occur. The lesion is always in the hemisphere opposite to the hemi-spasm, and frequently rear the fissure of sylvius. It is often a gummy tumor. It is noticeable that like all other nervous symptoms of syphilitic contagion, these unilateral convulsions tend continually to change in their habits, and very often assume the common type of epilepsy.

Hystero-Epilepsy. Cases of epilepsy frequently partake of the character of hysteria, and deserve the name of Hystero-Epilepsy. Sometimes these cases are dependent upon syphilitic ulcerations, and erosions of the os uteri. In diagnosing this syphilitic epileptiform hysteria from syphilitic hysteria, we should remember that syphilitic epilepsy is generally the result of hereditary syphilis This is never the case with syphilitic epileptiform hysteria. Buzzard, who has written of these affections quite fully, and from an evident previous close study and large experience, believes that inherited syphilis plays an important part in the production of fits and convulsions of early life.

After the period of dentition especially, epilepsy in children, in whom epileptic heredity cannot be traced, should make us suspicious of hereditary syphilis. In cases occurring before this period it is well to remember the criticisms of Hughlings Jackson upon a case of so-called syphilitic convulsions in a child a fortnight old

I. That convulsions are so common in children of healthy mothers that there is no great probability of syphilitic causation.

II. It is impossible to determine whether in children of this age the fits be epileptic or eclamptic.

Epilepsy. This form of syphilitic disease usually is wanting in an aura, the initial epileptic cry, the foaming at the mouth and the somnolent condition. Consciousness may exist to any extent from, perfect consciousness to the absolute loss of it, so common in genuine epilepsy it may also in the length of time, during which unconsciousness exists. Syphilitic epilepsy differs from the true or non-sphyilitic variety in the characteristics of the intervals. In true epilepsy we find usually that the patient during the intervals is comparatively free from the disease. In this type of the affection, however, these are during the intervals—nocturnal headaches (always a symptom indicative of syphilitic disease) osteocopic pains, insomnia, frequent attacks of petit mal or convulsive twitchings of the eye, or a limb, or merely vertigo, or faintness, extreme unaccountable nervousness and apprehension. Syphilitic epilepsy and its concomitant symptoms are especially prone to relapses. These epileptic attacks often precede the mental symptoms; or they may exist in connection with mental disturbances, and are then usually accompanied by complete unconsciousness, and are generally complicated by paralysis of cranial nerves. Dr. Wilks (Guy's hospital reports, series iii, vol. xvii,) says that any departure from the usual symptoms of a true epileptic attack should excite our suspicions as to its nature, and suggest some specific exciting cause for it.

Sensor Accidents of Syphilis.—In speaking of syphilitic paralysis we have called attention to irregular anæsthesias, which complicate that affection. We may also have anæsthesias of individual cerebral nerves complicating syphilitic mental disease; or instead of this condition of anæsthesia we may have the direct opposite hyperæsthesia of the cerebral nerves. A hyperæsthetic condition of the scalp should also be noted as occurring in constitutional syphilis, which is highly destructive to the hair. The headache which occurs in syphilis is generally intense persistent, and accompanied by nocturnal paroxysms. Pains which cannot be charged to anything but syphilis, "course over the scalp, pierce the ribs, scrape the skin, drag at the shoulder blades and loins." In syphilitic fever, frontal headache is a very common symptom. It is of great importance as a symptom in syphilitic epilepsy—it is one of the most common and immediate prodromata of the convulsion, but also occurs during the intervals, as we have noticed in speaking of syphilitic epilepsy. In cases of syphilitic deposit in the brain, headache accompanied by tenderness of the scalp is scarcely ever wanting—it is in such cases more intense than in non-syphilitic tumors, especially if they be of the membranes. Headache also appears in syphilitic mental disease, and at the beginning may be quite diagnostic, if it be possessed of the specific symptoms. After the development of syphilitic mental disease, it is more common in company with anæsthesia of particular spots, than neuralgia, which is likely to be found in connection with non-syphilitic general paresis of the insane. It must be remembered specially in the cases of children, that insomnia is often a symptom of headache.

We must here draw attention to a phenomena spoken of by Weir Mitchell; he says, "I wish also to call attention to the curious swellings like urticaria, which followed the attacks of pain; these also, according to my experience are seen only in cases of pain in the head, probably due to local meningitis of specific origin." Cranial pains are very frequent in constitutional syphilis—they are usually situated in the frontal region, and are more severe at night on account of the warmths of the bed, they are usually increased by pressure, and are constant in position. In connection with mental symptoms, these pains are strongly suspicious of syphilis, however, they are not always present in syphilitic patients, and Vanker Dolk says that cranial pains with nocturnal exacerbations, increase of pain on pressure are also seen in patients who have never been suspected.

NEURALGIA.—Dr. Anstie in his work on "Neuralgia and the diseases that resemble it" says, "and as regards the whole relation of syphilis to neuralgia, I must, from my experience conclude that the former is after all but rarely concerned in the production of the latter. Syphilis has a strong specialty for producing limited motor paralysis, but a much weaker one for producing limited affections of the sensory system." Eulenburg says "syphilis may be the direct cause of neuralgia, either by the development of specific gummata in the nerve trunks, or in the nervous centre, or by arousing chronic interstitial processes in the nerve sheaths, the membranes of the brain and spinal cord, or especially in the bone and periosteum." (This last mode of production is not a common way—periostitis produces a peculiar nerve pain differing from neuralgia, and of which we shall have more to say hereafter.) Syphilis may produce neuralgia by exciting inflammation of tissues, and pressure upon the nerve without, however, implicating the nerve itself in the inflammatory processes. When syphilitic tumors of the meninges encroach more or less upon the pons varolii, neuralgia is a prominent symptom, not unfrequently attended by deafness of one or both ears, of variable intensity. "Towards the close of the 15th century a great epidemic, believed to be syphilis pervaded Europe, * * * the historians of the disease described a form of neuralgia as one of the remote results of the venereal poison." (Aitken's. Sci. And. Pract. of Medicine.) There is a variety of neuralgias which Clifford Allbutt describes as functional—they occur in both the superficial and visceral nerves, they are pure and uncomplicated, not often fixed and do not return to the same place, perhaps may change at each paroxysm; often the patient cannot himself define their position. Dr. Broadbent does not think pure neuralgia to be very common. They are generally neurotic patients, in whom neuralgia appears, and syphilis is one of the existing causes which may draw out the tendency. Pure neuralgias are usually secondary, but may even precede the first eruption. They often alternate with cranial pains in mental disease from syphilis. Closely allied to the neuralgias are the radiating pains of periostitis affecting the cranium (preferably the anterior portion) and their cartitages, the sternum, tibia and other long bones. They are much more common in hereditary than in acquired syphilis; appearing from fifteen to twenty days after the appearance of the infecting chancre, or some days prior to the appearance of secondary cutaneous and mucous affections; they may disappear spontaneously, but they do so much more rapidly under the influence of mercury, iodine and local antiphlogistics. Syphilitic superficial neuralgia may be diagnosed from periostites, by the fact that it is neither aggravated by warmth, nor is the pain nocturnal.

Migraine is not an unfrequent concomitant of constitutional syphilis. Anstie relates a case complicated with dimness of vision, and paralysis of muscles of the eye, especially the levator palpebrae, and of the external recti, when the neuralgia rapidly disappeared under the influence of iodide of potassium daily, the paralysis was much slower in disappearing. Many authors have noticed a double sided prosopalgia (facial neuralgia.) Neuralgia of the trigeminus is particularly likely to complicate syphilitic mental disease, and is often followed by anæsthesia of the same nerve. Anstie says that syphilis appears as the cause of sciatica with unsuspected frequency. Of the visceral neuralgias, those of the stomach and heart are the most important. Gastralgia is not uncommon, while palpitation and angina pectoris as the symptoms of cardiac neuralgias are quite common. Anstie sums up as follows: "Supposing the above symptoms to be present (referring to such symptoms as would most probably occur in a case of genuine neuralgia) we expect to find * * * hereditary neurotic predisposition * * * or malarial blood poisoning * * * or a sufficient peripheral irritation * * or a constitutional syphilis. In this case there will be marked syphilitic local affections of the trunks of a nerve, or as is more common, the syphilitic change is in the nerve centre, there will most likely be other syphilitic centric mischiefs, leading to scattered motor, or vaso-motor paralysis, characteristic modifications of special sense, functions, &c.," There are two kinds of syphilitic pains to be distinguished from neuralgia, arising from syphilis. 1st. Those occurring early in the constitutional affection (dolores osteocopia) coincidently with or just before the secondary skin eruptions. 2nd. Those occurring in the tertiary stage, and are the immediate precursors of the formation of periosteal nodes. The second class of symptoms are particularly liable to be mistaken for neuralgia, when they affect a single spot of one side of the scalp or face. I must also speak of the pains produced by nodes upon the internal surface of the cranium, they are usually of a very intense character, and are mostly continuous, though aggravated from time to time, especially at night. Syphilitic meningitis is very generally diffused, and produces alarming symptoms. It is, however, some times more limited, when pain may be for some days the only noticeable symptom. There is a deafness dependent upon inherited syphilis, which was first remarked by Mr. Jonathan Hutchinson in 1863. Upon examination of the ear nothing can be detected in the external and middle ear to account for the loss of hearing However, evidence of inherited syphilis such as the typical teeth, and interstitial keratitis may commonly be found. Mr. Dalby in his lectures at St. George's Hospital, London, says that he has observed it as early as the fifth year, and never later than the twenty third year. Generally it is very rapid in its course, but may vary considerably. That the disease exists in the nervous apparatus is evident from the fact that the tuning fork is not heard through the cranial bone, and from tinnitus aurum; it is generally symmetrical. Treatment is useless. In this connection it is worth remembering that there is a deafness which often complicates secondary syphilis. Here the tuning fork is heard or not without regard to its position. Hearing in this case usually returns with the recovery from the constitutional disease.

INTELLECTUAL DISTURBANCES OF SYPHILIS.—Mental symptoms may occur two months or even two weeks after infection, and certainly with the first exanthema or general glandular swelling. Indeed cases have been reported where roleola, ulceration of the throat, and other symptoms of syphilis occurred secondary to the mental disease. Dr. Otto Braus (Psychiat. Centralblatt, No. 3,) in dealing of brain syphilis reports forty-five cases in one hundred as presenting spmptoms of insanity. In one thousand and ninety seven insane patients five hundred and one males and five hundred and ninety-six females, Dr. Wille found syphilitic causation in forty-six men and twelve women, i. e. nine and a quarter per cent among men, and two and a half per cent among women. This is a small proportion, because these statistics are taken from institutions admitting a rural population; because syphilitic mental disease is more likely to be treated privately or in general hospitals than in insane asylums, and finally because of the difficulty in procuring confessions of infection

Griesinger, speaking of these disturbances says: "This condition especially affects individuals whose brains are organically troubled, who have previously presented symptoms of abnoomal cerebral activity, or in whose families nervous disease has frequently occurred." It often exists, however, when no such predisposition exists. A renowned French alienist declares that one-twentieth of the female lunatics at the Salpetriere have been prostitutes. And here it is proper to take note of a statement by Pritchard, which has some bearing on the question in hand, he says that women of the Vallais are much more apt to have cretin children when married to Savoyards, who are commonly debauches, than those who marry the more virtuous mountaineers from the higher Alpine country. Connected with the early nervous symptoms of syphilis we may have a series of so called functional mental symptoms, restlessness and excitability almost maniacal, or more or less prolonged stupor. Very often a slight obscuration of intellectual functions is observed. There may also be mentioned under this head the cases of mental atony, so often observed in early syphilis, and followed by torpor, and finally prostration. Hypochondriacal delusions with special reference to the incurability of an infection may present themselves during the earlier stage of the disease. They may be said to suffer from syphilophobia. If syphilophobia presents itself in several degrees of severity, one patient is satisfied and indeed cured (as far as mental symptoms are concerned) by the administration of aqua coloratum, ad libitum, with the assurance of its efficacy. A second, however, is furious, suicide is very apt to be the termination of the case if strict watch is not kept upon him. These patients are very likely to have all the diseases in the books, and every minor symptom is enlarged until it assumes the proportion of a very fatal indication. Mental disturbances in the form of hypochondria generally are the first manifestations of cerebral syphilis. The hypochondria may be replaced by forgetfulness or maniacal attacks, or even the monomania of grandeur, and sometimes by progressive dementia with marked loss of memory, but without the large ideas. Insomnia generally complicates hypochondria. Melancholia often is present among the nervous symptoms of syphilis, markedly during syphilitic fever. Dementia occurs usually in the later stages of syphilis, it is usually progressive, with the monomania of grandeur and loss of memory. The rapid decline of the mind into dementia is almost characteristic, and distinguishes it from the common secondary form of that disease. It sometimes alternates with mania or alteration of character. The duration of the syphilitic mental disease is variable, lasting from one or two weeks to several years.

Diagnosis and Symptomatology.—Syphilitic neuroses may appear with the first secondary symptoms, or even two weeks or months after, even before the appearance of the first exanthemata, or with the general glandular swelling. The disease is not infrequently lighted up by injury. (J. Hughling Jackson—Tourn. Mental Science, July, 1874.) The paralytic seizure, when dependent upon syphilis, is generally the immediate result of some violent exertion, or long continued muscular effort, carried on to fatigue, and the collapse is often so great as to threaten immediate death. The course of syphilitic affections of the nervous system is often marked by a certain variety, by a change in the symptoms. Some imes the most severe and threatening ones rapidly disappears, and give place to a comparative quiescence; or sometimes intellectual, sometimes convulsive, and sometimes paralytic symptoms take the upper place. Functional disorders of digestive organs, and of the circulation, and accompanied by vague nervous sensations are very frequent in the course of any syphilitic nervous affections. In non-syphilitic cerebral disease, nausea and vomiting are very common at the commencement, but in cerebral syphilis, it is extremely rare In diagnosing, suspected syphilitic affections of this character, careful and full examinations into the previous history of the patient must be made—his social position must be considered. The word of the patient is not always to be taken as conclusive of his freedom from specific disease. Scars of old chancres, &c., are of course invaluable as evidence. However, when the history of primary syphilis is found, we should carefully examine for sequelæ of primary disease, and the presence of any constitutional taint still existing; but remember that neither a resultless examination, nor the absence of the secondary symptoms can gainsay the existence of the disease. We must enter all the more thoroughly into an ex.

amination of the series of symptoms, when they raise even a suspicion of a syphilitic origin. Indeed, Weir Mitchell calls attention to the fact that "syphilitic neuroses seem most apt to occur in persons who have had no eruption, or other evidences of constitutional disease." Dr. Broadbent, in the Lettsomian lectures (Lancet, March, 1874) says: "I have formed an opinion, that it is chiefly in persons in whom the secondary affections have been transient, and insignificant, or even absent, or in those in whom the tertiaries arrive early or primarily; that the nervous system is liable to suffer." This is often the case with the usual tertiary accidents, and should be considered, when an attempt is being made to trace a syphilitic history. In women, the primary lesion is so hidden and the discharges so changed by the normal ones that it may be unperceived. Again the syphilis may have been communicated by the foetus, and so the history of the primary and secondary stages be entirely absent, and perhaps this is the best place to warn you against the conclusion that everything which occurs in a person who has suffered from syphilis, is due to that disease. I have already spoken of the age at which these affections may be expected —early manhood or early middle life. In childhood, of course, hereditary is to be considered. In old age, however, we should come to the diagnosis of syphilitic disease only, after the exclusion of other and more common disease. The random association, and succession of phenomena, to which attention has been drawn already, is usually well marked. Mental symptoms alternate with motor, and sensor disturbances, or all three may for a longer or shorter time be associated, when suddenly, and without warning, new combinations of symptoms are established, to be again changed in an equally sudden and unexpected manner. Paralyses of cranial nerves and other accidents, serve to render these arrangements of symptoms more irregular. Behrand says that when no other cause can be found to explain a pathological cerebral symptom, syphilis, in consequence of its frequency may be accepted. Dr. Moxon believes it to be incumbent on every one who has a case of local intracranial disease under his care, to treat it at once with iodide of potassium, without waiting to make out its character. Bedel says that if specifics do not act, there can be no syphilis.

This, however, is somewhat too strong, as I hope to show, when speaking of treatment. The tendency to relapse is frequently of importance as a diagnostic sign of syphilitic nervous disease. Mr. Jonathan Hutchinson believes that tertiary syphilitic symptoms are generally asymmetrical. Cases exhibiting symptoms which cannot be referred to any one localized lesion of the nervous axis should be carefully examined for syphilitic taint, for it is seldom that any cause, save syphilis, produces two lesions of the nervous system unconnected, save by their similar cause. The existence of marked cachexia unexplained by evident disease of any of the visceral is of service in pointing to syphilis, as the cause of concomitant phenomena. Prolonged insomnia in adults or children should lead to grave suspicions of syphilis. In alcoholism the mental symptoms differ from the melancholia and dementia of syphilis, inasmuch as they are by dreams, illusions, and even hallucinations. Consciousness is seldom affected, while sensibility is usually disturbed. In lead poisoning, the paralyses sometimes simulate syphilitic paralyses, but the fact that the former are almost invariably limited to the extensor muscles of the limbs, should serve to distinguish them. Tubercle or sarcomata must be excluded by the age, morbid antecedents, and the modes of evolution. Cerebral softening is followed by a persistent hemiplegia, which is but little subjeet to amelioration. Pachymeningitis, and non-syphilitic tumors of the dura-mater affections, which most closely resemble, symptomatically, syphilitic epilepsy, &c., are distinguished by the rapid and almost sudden appearance of acute symptoms, generally characterized by somnolence and other signs of compression, the other by a cephalalgia, in general little intense, and symptoms slowly progressive; but besides, these affections do not present the character of the syphilitic lesions; they are exempt from the cachexia, peculiar to individuals who have reached the visceral period of the latter disease.

General paresis deserves a few words, while speaking of the symptomatology of these affections. Erlenmeyer believes that general paresis is a syphilitic process, that in every case an earlier syphilitic infection has existed, but admits that the connection can rarely be demonstrated. Iessen, (1857) also believed in the syphilitic origin of general paresis, and claims to have confirmed this condition in almost all the cases observed by him. Wille does not believe that the statistics of insane asylums favor the identity of general paresis, and cerebral syphilis. It is certain, however, that symptoms similar to the phenomena of general paresis, often succeed to the ordinary mental symptoms of syphilitic cerebral disease. Syphilitic progressive dementia occurs generally between the ages of twenty and thirty years, it is usually complicated by the appearance of paralyses of cerebral nerves, most frequently blepharoptosis and strabismus. Paralyses are gradual in their origin, and often pass away in a few days, but generally last for sometime.

The most common form is hemiplegia, it often follows apoplectiform attacks. Quite frequently we have epileptoid attacks. Anæsthesia is quite common in syphilitic progressive dementia, as also is headache, which is usually noticed at the commencement, and is quite intense. Curability is generally the feature of the syphilitic psychosis, but the more the symptoms simulate general paresis, the prognosis becomes more unfavorable. The pathological changes of the syphilitic progressive dementia are usually localized, as I shall show you in the rule in syphilitic cerebral disease, when I come to speak of pathology. And now a few words as to the diagnosis between this syphilitic psychosis, this false general paresis, and the genuine non-syphilitic general paresis.

Primarily the patients in the genuine affection are usually older than in the syphilitic affection. They are generally troubled by neuralgic pains, rather than the cephalalgia, and anæsthesia of the syphilitic patients. Paralyses generally occur without warning, and pass off very rapidly. Probably the most distinctive feature, however, in the appearance in the syphilitic disease, is paralysis of cerebral nerves. Wille does not think that the two affections can be diagnosed in any other way than by the appearance of these paralyses. Epileptoid attacks are seldom observed in general paresis. They are most frequent in syphilitic psychosis. General paresis is almost always fatal, herein differing again most materially from the syphilitic disease. Again its pathological changes are contrary to those of syphilitic progressive dementia, the latter are often multiple, the former, never so, although sometimes quite general. In cerebral syphilis, as indeed in almost all syphilitic nervous diseases, polyuria is quite frequent.

We come now to speak of the prognosis of the nervous accidents of syphilis. Individual cases must be judged by their particular symptoms, but the following are a few general observations; uncomplicated primary mental disease is not unfavorable, the worst prognosis being, when these mental symptoms assume the likeness of general paresis of the insane. Gjor (quoted by Lanceraux) reports that in thirty cases of cerebral syphilis, five were cured, twelve relieved, six obtained no relief, and seven died. Lagneaux, Jr., (also quoted by Lanceraux) reports one hundred and forty cases, eighty-three cases terminated, more or less unfavorably, and fifty-seven were fatal. These figures are by no means fair. They are cases of the worst character, which are here drawn together, and hence the unfavorable reports. Those nervous affections which are termed functional, are much more stubborn to treatment than "organic" cases. Dr. Clifford Albutt "on the obscurer neuroses of syphilis" West Riding Lunatic Asylum Reports, Vol. III, says: "I much regret to say that my own experience of positive nerve lesions in syphilis, is much less comfortable than that of others would seem to be. Promptly they may recede under appropriate remedies, but they recur again and again, to be driven out less and less easily." Destruction of tissue from any cause, or of any description, of course, renders the prognosis extremely unfavorable, but even in such cases, amelioration almost amounting to cure not unfrequently occurs. Again the more disseminated the lesion the more unfavorable becomes the lesion. In intra-cranial growths, the prognosis is comparatively favorable, the most unpropitious site being the duramater. We should always remember the transient and mobile character of these growths, and not mistake a temporary improvement for a permanent relief. In paralysis of the cerebral nerves, the prognosis is almost bad. Paralysis of muscles of the eye ball is seldom permanently relieved. The longer the duration of the disease, the less the chance of recovery. Each succeeding attack renders the prognosis less favorable. Beware of latent disease after apparent recovery. Cephalalgia and insomnia have no serious import. Vertigo and convulsions are not always decisive in their value, but are less formidable than paralytic or psychic symptoms.

Pathology.—Almost every leison of the cerebro-spinal axis has been found in connection with syphilitic disease of these organs, and besides, there are one or two characteristic leisons of syphilis. Often however, several of these leisons are found coexistent without any marked symptoms having been noticed during life, resembling in this respect the habits of malignant disease. Syphilitic changes generally attack only a small part of the organ and the remainder is left quite free. You will remember however, what I have said about multiple leisons. Children are not unfrequently the victims of this disease, and all of these leisons may be found, but the difficulty, or rather impossibility to render a perfect diagnosis of such cases from the effects of tubercular meningitis, has prevented anything like a satisfactory study of their phenomena. There are three lesions of almost diagnostic character, syphilomata, exudations and cerebral thrombosis. The gummy tumors which are so characteristic of syphilitic disease seldom exist in the brain alone, but are found in other organs, more frequently the testis and liver. It is not easy to mistake a gummy tumor. It is strictly specific, and has its favorite seat in the periphery of the encephalon chiefly in the region of the anterior or posterior lobes of the brain. These tumors are generally multiple, and often grouped and joined together by fibrous bands or strata of a whitish yellow color, and vary in size from that of a pea to that of a walnut, and are occasionally surrounded by a patch of inflamed tissue. They are usually composed of two distinct portions—an outer fibrous and an inner gummatous or even casseous substance. The outer fibrous shell is smooth and more or less adherent—it represents the normal fibrous supporting elements of the part, in a state of augmentation—the "functionary" elements being degenerated and atrophied. This may comprise the entire growth, no central part differing from this can be found. But generally there is to be seen in the centre, sharply distinguished from the fibrous outer part the now softened casseous or gummatous faint yellowish matter of more or less firm consistency. Sometimes it is firm and elastic, at others curdy, and again it may calcify. Very often these contents are absorbed and the outer portion alone left, and we are then in danger of mistaking them for cerebral cysts. In constitutional syphilis there is a tendency to the effusion of a low form of lymph or fibro-plastic material. The special exudations of cerebral syphilis are but specimens of this action in the cranial cavity. They occur most frequently upon the meninges, and specially the dura mater, although they may be found upon the cerebral cortex and cranium. The interpeduncular space at the base of the brain is the favorite site for syphilitic exudation. They generally occur during the tertiary period, are generally multiple and appear as small circumscribed yellow deposits surrounded by more or less inflammation and softening, under the microscope they present the characteristic small cells, fatty granules and some amorphous matter. This small celled character is specific, although there be no growth. The specific character is much more certain if these foci of deposit are distant from the common position of the pacchionian granulation. Syphilitic cerebral softening may be of two kinds. 1st, that which is the result of syphilitic thrombosis or other disease of the cerebral arteries. 2d, that which is consequent upon an encephalitis, which in its turn may be idiopathic depending upon the syphilitic cachexia for its causation or from mechanical irritation such as would occur in the immediate neighborhood of a gumma, or from the encroachments of a meningeal or osseous growth. "The anatomical characteristics which distinguishes syphilitic cerebral softening from softening of the brain from obliteration of arteries, would be the absence in the latter of any new formation," *Lanceraux.* This statement is hardly correct, and I read it simply to show you that new formation is not the only characteristic of syphilitic disease. Softening not depending upon disease of the artery, may present no new growth, and new formation may exist in connection with, and as the cause of obliterative disease of cerebral arteries. Lanceraux (annales de Dermatologie et. Syph.) describes the spinal cord of a still-born child of syphilitic parents with condition of liver described by Guoler,—the cord was reduced to a mass of neuroglia with scattered oil granules—no traces of ganglion cells or nerve tubules could be discovered.

Meninges unaffected, and a twin child showing unmistakable signs of syphilis, presented no alteration of the cord.

Dr. Petrow, of St. Petersburg, has lately published the results of his examinations by the microscope of sympathetic preparations being taken from the cervical thoracic and solar plexuses, ten, twenty and twenty-four hours after death. The following is simply a synopsis of his published results. 1st, small refracting pigmentary granulations, either disseminated or confluent, increasing in number with the duration of the syphilitic cachexia, and finally completely masking the nucleus and nucleolus. These may be partially dissolved by nitric acid and potassa. The endothelium is occasionally the seat of a very abundant cellular proliferation—pigmentation of nerve cells have been several times observed, which could be traced to no pathological condition, but occurring in old age, the granulations are however isolated, and the nucleus always visible. 2d, A culloid degeneration of the nerve cells and endothelium—the protoplasm presenting a brilliant homogenous mass which swells up and is rendered opaque on the addition of acetic acid. 3d, Interstitial lesions which are most numerous,—manifest hyperplasia of interstitial cellular tissue, forming cellular bands, which appear to dissociate the cells and nerve tubes. The cellular elements participating become opaque, and finely granular contours are effaced. The nuclei however remaining visible. In long standing cases these conditions of the endothelium are found to have degenerated into finely granular masses, soluble in ether. The nerve cells become angular, and the protoplasm pigmented; nerve fibres are compressed, their envelopes are thickened, the myeline sometimes changing to granular detritus. Dr. Petrow in his examination, used a solution osmic acid, (1 pt. to 400 or 500 pts of water). After macerating the preparation in this solution for five or seven hours, the nerve elements were stained black without modifying the connective tissue.

Diseases of the Cerebral Arteries.—Thrombosis is the most frequent lesion, although embolism and other changes may occur. Thrombosis may be caused by a diseased or altered condition of the blood, by a lesion of the arterial walls, such as a gumma or exudation, or both of these causes may be combined in the production of the thrombus. Sometimes a simple inflammation, an arteritis may occur producing a thrombus; in such cases the inflammatory action usually begins in the outer coat; another mode of the production of arterial obliteration, especially in the pia-mater, is produced by the meningeal exudations binding the membranes to the convolutions, occluding the arterial canal by means of compression. Indeed thrombosis of the arteries of the base are generally accompanied by thickening adhesions of the dura mater in the neighborhood. Syphilitic deposits may occur in any one of the arterial coats, or in the perivascular space; in the latter case the proper tissues are either absorbed or atrophied and indurated, while this specific exudation occupies its place in the form of a transparent granular matter sometimes almost of a greenish tinge. When the disease affects the arteries themselves, which is less frequently the case, the outer coat is at first infiltrated, this extends gradually to the inner coat, and then the calibre of the vessel is diminished by the protruding deposit while the presence in the other coats interferes further with the nutrition and function of the artery. The arteries generally appear enlarged and of irregular contour, and on account of the disappearance of the intermediate nerve substance in close proximity. The disease occurs in well defined foci not necessarily connected. After this condition of diminished arterial calibre has become well established, occlusion may be expected at any moment from a blood clot at the point of constriction and neurotic softening of the part of the organ dependent upon the diseased vessel for arterial supply. Cerebral softening from syphilitic thrombosis has no particular characteristics over the non-syphilitic variety.

Lesions of the Meninges.—It is worth noticing as a fact of diagnostic and pathological importance that the dura mater is the most frequently affected. In speaking of meningeal affections, we may mention syphilomata, adhesions and inflammation. Syphilomata may be found on the pia mater, and extending to and affecting the gray substance, and particularly along the course of vessels or upon the dura mater. They generally excite adhesion; 1st, of meninges to the brain tissue. 2d, between the meninges and, 3d, between meninges and bone. Griesinger declares a partly diffused, partly circumscribed thickening of the meninges to be specific. Verdun, Meyer and Maudsley, do not consider the simple exudations as specific without these adhesions also exist. The adhesive material is adventitious, of firm consistence, harder, tougher, and more opaque than lymph, of a yellowish color. The meningitis following the erratic course of all the syphilitic nervous phenomena exists generally in places in which it is much more rarely seen in non syphilitic meningitis. The basilar meninges, and especially the neighborhood of the cerebral nerves is a favorite site. In the brain tissue proper, we find almost any lesion. Anæmia and hyperæmia single, may be diagnosticated in connection with any syphilitic nervous disease, especially mental disease. Anæmia is much more common than hyperæmia. Encephalic foci of interstitial encephalitis, have been described by Virchow, and are not uncommon in constitutional syphilis. Softening—this is also usually constitutional,—I have already described how this condition may be produced by disease of the arteries. It may also arise from the impinging of meningeal growths upon the encephalic tissue. Again, it may follow upon lesions or growths of the tissues themselves, such for instance as encephalic exudations, or by syphilomata, and finally cerebral softening has been noticed where no cause could be assigned, save the syphilitic cachexia. Prof. Alonzo Clark, of New York, reported several years ago, the case of Dr. David Green, who became infected during the labor of a woman of suspicious character, and dying from the effect of paralysis, &c, which developed during the progress of the case. Upon post-mortem, a cerebral softening was revealed. Encephalic induration may also occur as a tertiary accident. Virchow reports the case of an army officer, who had long been suffering from constitutional syphilis, and who during the latter part of his life, has pain and stiffness in the neck, paralysis &c., on section he found the longitudinal sinus intact, and the brain depressed at its cavity, the convolutions which were flattened and small, contained but little blood; the cerebral substance which was yellowish and very tenaceous, almost resembled leather in consistence. The ventricles of the brain were filled with serum, (Lanceraux, p. 48, vol. II. Sydenham.) Syphilomata have been already described. In children and adults suffering from syphilis, cerebral hemorrhages are not uncommon. Meningeal growths may affect the encephalic mass in two ways, by producing adhesions, and by thus occluding the vessels of the pia-mater, producing lessened blood supply to the peripheral gray matter, and consequent atrophic changes of some kind, or they may impinge directly upon the nervous tissue, and by pressure produces various lesions, usually softening. I am constrained to believe with Dr. Moxon, that the surface of the Medulla Oblongata is the favorite locality of syphilitic lesions, rather than the substance as claimed by Dr. Broadbent. Chronic hydrocephalus often occurs as one of the effects of congenital syphilis. The spinal cord is also affected by syphilis, much the same as in the intra-cranial contents. Myelitis and exudations are the most frequent lesions. Myelitis may be acute, general and local—sub-acute and chronic. Lesions of the bones in syphilis are principally of two kinds, gummata and exostoses. The latter, usually from about the frontal sinuses, and on the long bones at the region of the epiphyseal cartilages—all these facts going to prove that the positions of late developement are unusually liable to disease. And now a few words as to the treatment of the effections. I must warn you at the outset of the very agreeable theory of specifics. Many persons speak as if the only necessaries to the successful treatment of any syphilitic nervous disorder, is plenty of Iodide of Potassium and mercury in one form or another. If you confine your medication to these or any other so called specifics, you will utterly fail. Every symptom, not only of the nervous system, but of each and every organ and system must be carefully studied in all its bearings, and remedies applied only after and in view of such examination. The truth is, and strange as it may seem, it is seldom fully appreciated, that we need as a menstruum for these specifics, what Opie advised the young and inquiring artist to use in mixing his paints—Brains!

Nor would I have you think that there is no efficacy in the exhibition of mercury and iodide of potassium. Dr. Broadbent, to whom we should give the greatest confidence, says: "Wonderful relief is often experienced, even after the lesion has lasted for some time by the removal of the primal cause." Dr. Buzzard, the latest authority, says. "I know of nothing in all therapeutics more extraordinary than the rapid effects of the iodide of potassium, in improving the condition of these patients, except it may be, perhaps the influence of lemon juice in scurvy." Perhaps the whole theory of the use of mercury, and the preparations of iodine may be stated in a few words. And just let me recall a few pathological facts—the principal changes—intra-cranial and intra-spinal, may be classified under two heads. 1st. Exudations and new growths, where there has been merely proliferation of cells. 2nd. Growths in which cell proliferation has been succeeded by fibrillation, and retrograde metamorphosis—syphilomata. The first group is the province of iodide of potassium, the second of the mercurials. So the rule of Dr. Broadbent is a safe one. "The one remedy is iodide of potassium, this failing—mercury." As to the use of the iodide, the same author says, "commence with doses of six or eight grains, combined with ammonia, either the carbonate or the aromatic spirits, and if no intolerance is developed, the doses may be pushed to twelve, eighteen, twenty-four, and even thirty and thirty six grains, thrice daily. The iodide should be given after meals. It is so diffusible a remedy that a continuous effect cannot be easily obtained, without some such precautions be taken." Dr. Buzzard—clinical aspects of syphilitic nervous affections, 1874. "There is something quite remarkable in the influence of the iodide of potassium, in cases of this class, the drug seems to act almost as food to the patient." Remember, however, that these are eminently scientific physicians, and are not accustomed to empirical medication. Iodide of potassium is not likely to be of use in hemiplegia, &c., but would be serviceable in cases of recent palsies of cranial nerves. When we can diagnose the lesions of the second class described above, mercury is undoubtedly our main stay, for it is their special antagonist. Dr. Hammond, Am. Journal of Syphilography reports that he has succeeded in removing the pain of syphilitic, neuralgia by bromide of calcium, when iodide of potassium, and mercury have completely failed. His perscription is as follows: ℞. calcu bromid. ʒi to ʒx aquæ q. s. ad. ℥is. sig. ʒi. morn. mid-day and ʒii. at night.

I have seen the effects of this treatment in one or two instances; but do not perceive any particular advantage. During the past autumn, I treated at the Dispensary, a case of quite severe trigeminal neuralgia of syphilitic causation. My treatment, subject of course to the variations in the phenomena of the case, is as follows: ℞. potassu. iodid. ʒiiss. chloral. hydrat. ʒiij. ext. cannabis. indicæ fluid ʒj. syr. tolut. ad. ℥iv. sig. ℥i. four times a day. One other point deserves mention before closing. In cases of hereditary syphilis, Tinct. Belladonnæ, in ten minim doses hourly is the best anodyne and sedative that can be given. I am indebted for this hint to Dr. George Thompson, of the Bristol asylum, England, who uses it in accordance with the teachings of Mr. Pridgin Teale.

373 Clinton Avenue, Albany, N. Y.

